# Nightmare after iliac vein stenting: Spinal epidural hematoma

**DOI:** 10.1002/ccr3.4522

**Published:** 2021-07-21

**Authors:** Yaser Jenab, Mohammad E. Barbati, Ali Ajam, Saeed Tofighi, Kaveh Hosseini, Houman Jalaie

**Affiliations:** ^1^ Interventional Cardiology Tehran Heart Center Tehran University of Medical Sciences Tehran Iran; ^2^ Vascular Surgeon European Vascular Center Aachen‐Maastricht University Hospital of the RWTH Aachen Aachen Germany; ^3^ Students' Scientific Research Center (SSRC) Tehran University of Medical Sciences Tehran Iran; ^4^ Cardiology Tehran Heart Center Tehran University of Medical Sciences Tehran Iran

**Keywords:** iliac vein, post‐thrombotic syndrome, spinal epidural hematoma

## Abstract

Spinal epidural hematoma is a rare but devastating complication of iliac vein stenting. Radicular back pain during and after procedure is an alarming sign for this complication.

## Graphical abstract

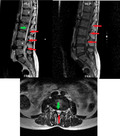

## 1 INTRODUCTION

In the present case, a 40‐year‐old woman developed secondary spinal epidural hematoma due to inappropriate wiring of the para‐spinal area during iliac vein stenting which presented primarily as acute back pain.

Iliac vein stenting, a minimally invasive percutaneous procedure, has transformed the management of the proximal deep venous occlusive disease. A retrospective cohort study in 2007 by Neglen et al reported primary and secondary stent patency rates of 57% and 86% for post‐thrombotic obstruction and 79% and 100% for symptomatic non‐thrombotic iliac vein lesions, such as May‐Thurner syndrome ^1^

The procedure is usually performed in the operating room or angiography suite with the patient under local anesthesia and intravenous sedation. Ultrasound‐guided cannulation of the femoral or popliteal vein is performed, followed by insertion of a sheath. Antegrade venography is performed to determine the degree, length, and site of obstruction, and the presence of collateral vessels. Once the area of concern has been traversed with a guidewire, The stenosis or occlusion is crossed with a stainless‐steel self‐expanding stent. Upon completion, a venogram is mandatory. If significant stenosis remains, post‐dilation with a balloon is carried out. Finally, the sheath is removed and light pressure is applied.^2^

Spinal epidural hematoma (SEH) is a rare neurological condition with challenging clinical management. This disorder can occur spontaneously or as a secondary condition and it represents less than 1% of space‐occupying lesions within the spinal canal. The incidence of spontaneous epidural bleeding is estimated to be 1 case per 1,000,000 populations per year.^3^ Coagulation and platelet disorders have been suggested as predisposing factors for SEH, with surgery and trauma regarded as other causes of SEH. It usually presents with acute severe back pain and signs/symptoms of spinal cord compression, mostly in the posterior segments. The spinal epidural venous network is the major source of the bleeding. Magnetic resonance imaging (MRI) is the imaging of choice, and urgent surgical intervention is required.^4^

## 2 CASE PRESENTATION

**FIGURE 1:**
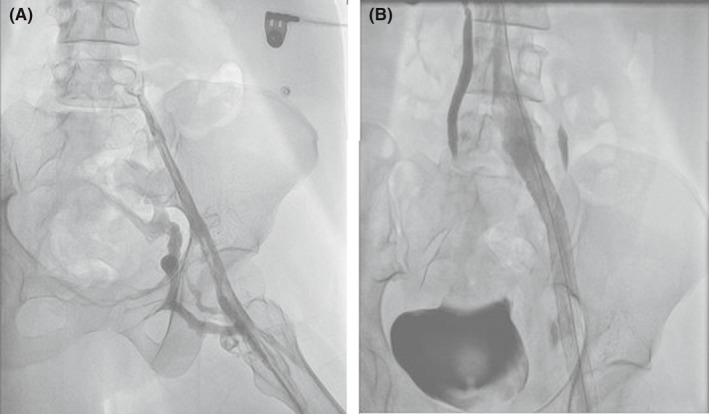
A, Venography showed occlusion of left common iliac vein, B, Common iliac and femoral veins stenting was done

**FIGURE 2:**
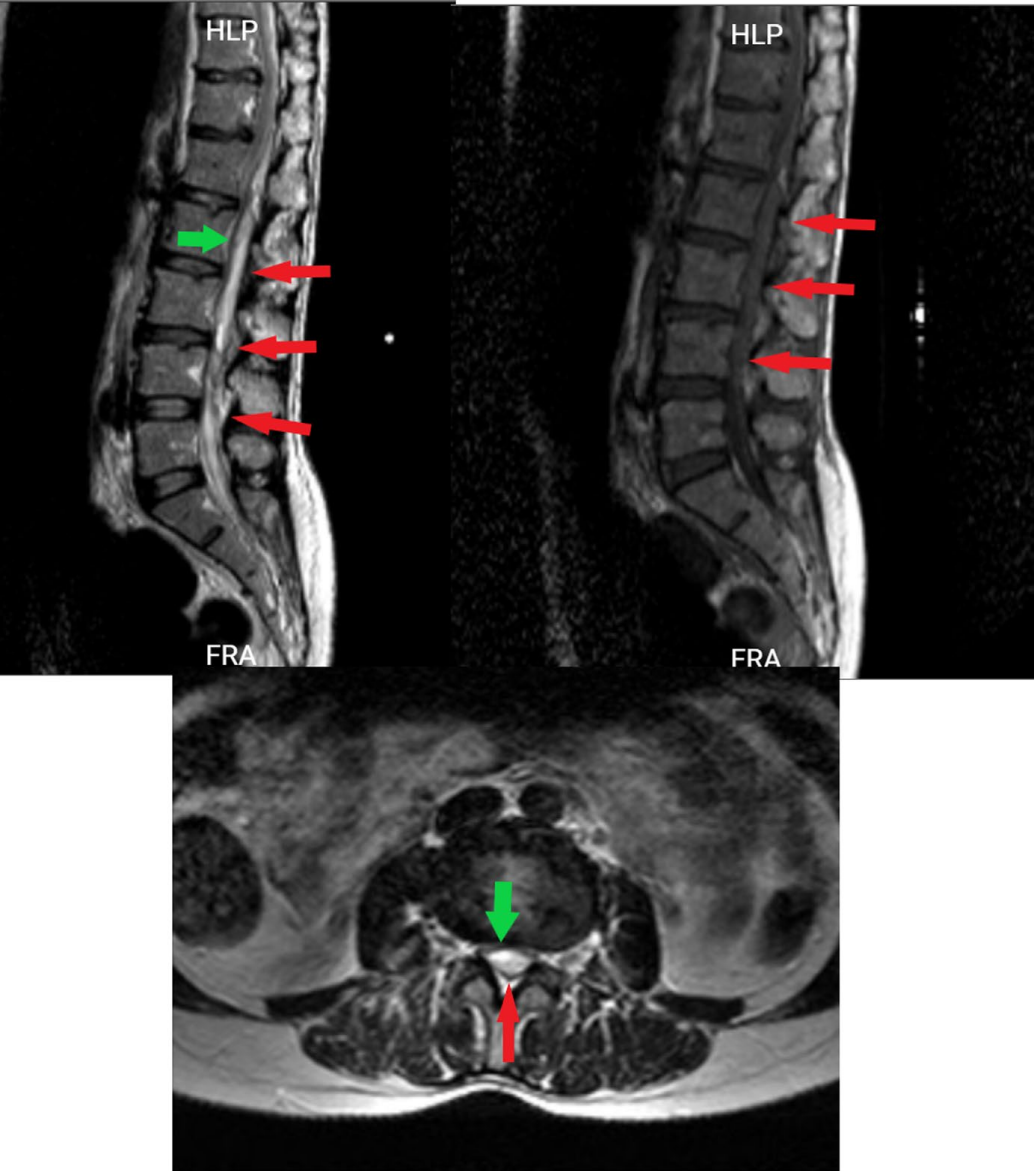
Lumbosacral sagittal and axial T2 and sagittal T1, red arrows represent hematoma and green arrows represent compressed thecal sac

**FIGURE 3:**
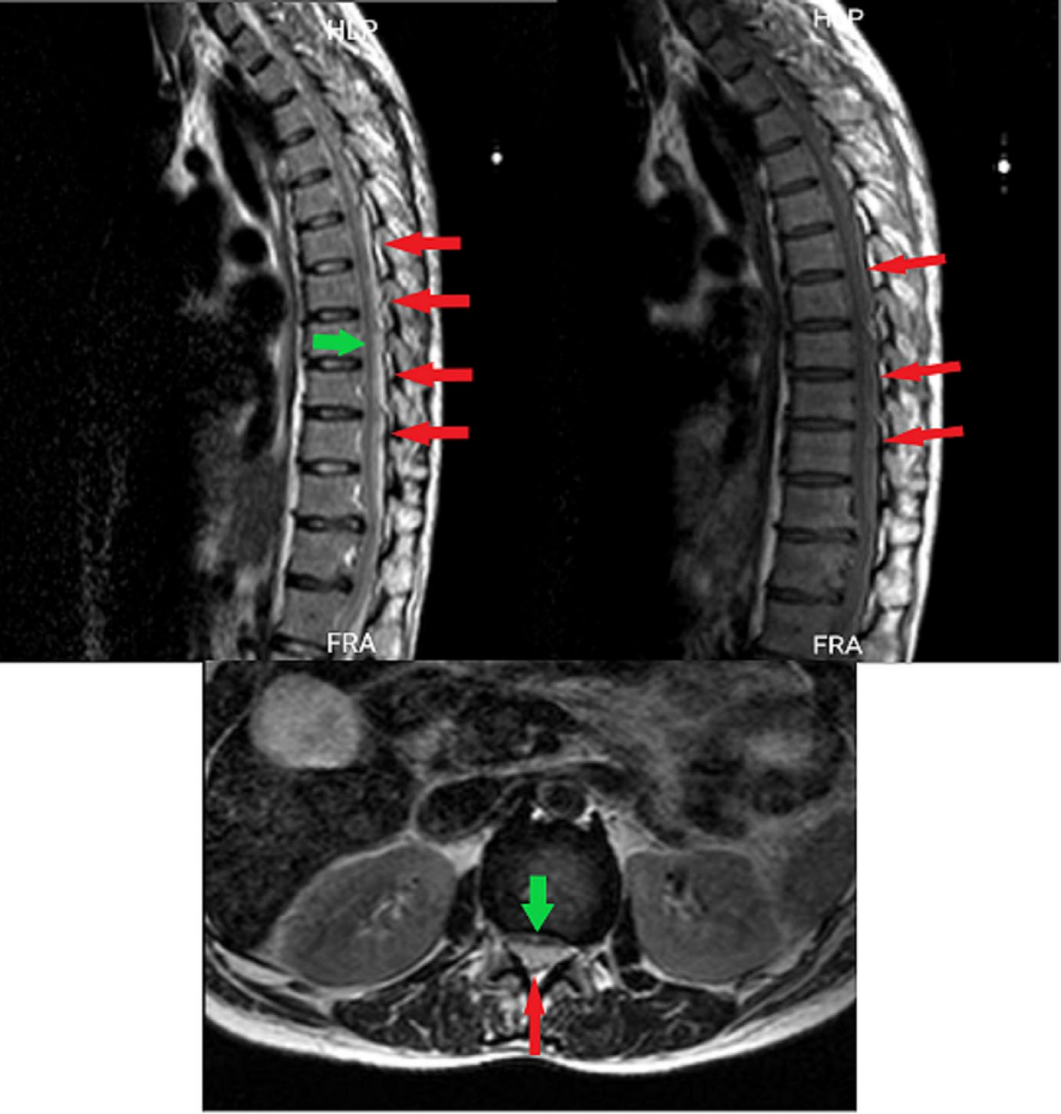
Thoracic sagittal and axial T2 and sagittal T1, red arrows represent hematoma and green arrows represent compressed thecal sac

**FIGURE 4:**
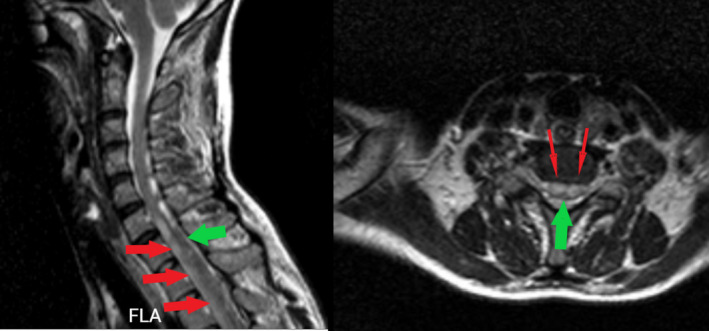
Cervical sagittal and axial T2, red arrows represent hematoma and green arrows represent compressed thecal sac

**FIGURE 5:**
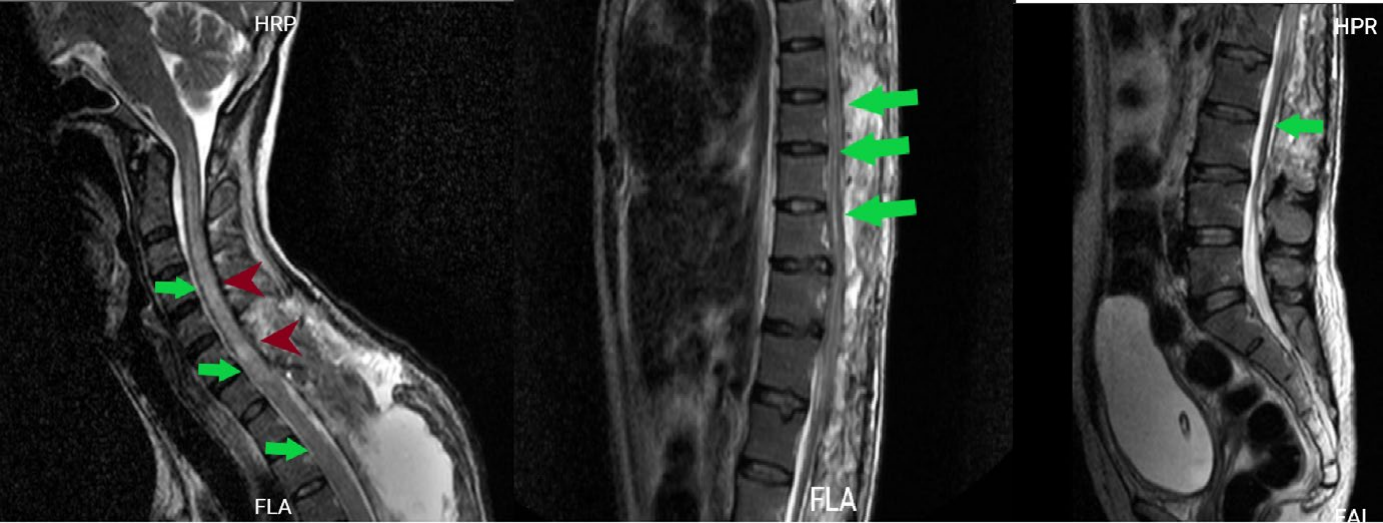
Sagittal T2 total spine after posterior laminectomy, dark red arrowheads represent compressive myelopathy in the cervical area and green arrows represent normally positioned thecal sac

A 40‐year‐old woman presented with the post‐thrombotic syndrome of the left lower extremity and a Villalta score of 12 points.^5^ She had a history of recurrent left lower limb deep vein thrombosis for approximately 10 years. Venography showed occlusion in the left common iliac vein compatible with May‐Turner syndrome (Figure 1A). Although the common femoral vein was partially occluded, the main inflow veins were intact. Due to significant symptoms which were unresponsive to medical management, venous stenting was planned. The patient was laid in the supine position, became anesthetized and a 10‐F sheath was inserted from the left femoral vein under ultrasound guidance (mid‐thigh approach). A hydrophilic stiff wire (0.035‐inch) and a glide catheter were used to pass the occlusion. The patient received 10,000 units of unfractionated heparin (UFH). After the confirmation of the inferior vena cava (IVC) cannulation, pre‐dilation with Atlas balloon (14 and 18 mm) was done, and 2 stents were deployed: a Sinus‐XL stent (18 × 40 mm) in the common iliac vein and a sinus‐venous stent (14 × 150 mm) from the left external iliac vein into the common femoral vein (Figure 1B). The Sinus‐XL stent was deployed as the proper stent with the specific size was not available on our shelf. The patient was on 1000 units per hour drip of UFH, to keep activated prothrombin time (aPTT) between 50 and 70 s. She received 300 mg clopidogrel bolus dose and 75 mg daily dose after the procedure. Twelve hours after the procedure, she complained of severe back pain radiating to the lower extremities. About 24 h after the procedure, she developed abrupt loss of sensory and motor functions in both lower limbs with progression to the upper extremities. MRI was done, which showed SEH at lumbar to cervical segments (Figures 2,3 and 4). Anticoagulation was discontinued and an inferior vena cava filter was placed. The patient was transferred for emergent laminectomy and hematoma evacuation. (Figure 5).

At 1 month follow‐up, the inferior vena cava filter was removed. Stents were patent but there was not much improvement in neurological condition.

At 3 months follow‐up, although there was an improvement in the neurological deficits of both upper limbs, no significant improvement was observed in the lower limbs.

**FIGURE 6:**
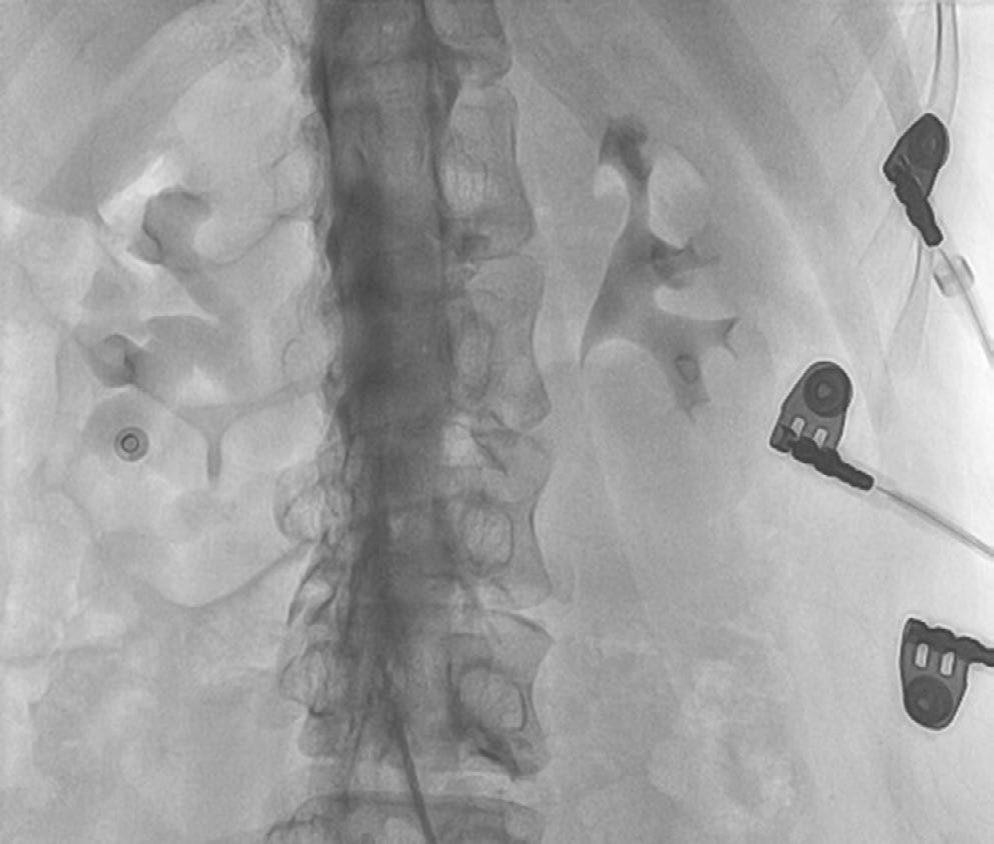
The image shows spinal epidural space enhancement during the index cannulation of the inferior vena cava

In a review of the videos taken during the procedure, we noticed the enhancement of the spinal canal with dye injection due to advertent wiring and cannulation of the spinal epidural space with the 0.035‐inch wire and the A1 catheter. Unfortunately, this was ignored by the operator presumably due to being unfamiliar with this rare incident (Figure 6).

## 3 DISCUSSION

Venous stenting for chronic venous obstruction has been developed as a suitable and less invasive therapeutic strategy comparing to conventional surgical bypass. In most studies, it had no risk of death, pulmonary embolism, and bleeding.^2^ Based on a study by Neglen and Raju, the early (<30 days) complication rate is low (10%).^6^ Bleeding, arterial cannulation, stent thrombosis, and in‐stent restenosis are among the reported complications of venous stenting.^2^ In general, the safety of this endovascular procedure is high, nevertheless, the experience of the operator plays an important role in reducing possible risks.

Lower back pain is also one of the complications during and after iliac vein stenting.^7^, ^8^ In a study by Zhu et al^8^ in 2019 on 45 patients who underwent iliac vein stenting, 11 (24%) and 10 (22%) patients experienced back pain during and within 30 days of the procedure, respectively. Although back pain is quite common in iliac vein stenting, it is imperative to rule out life‐threatening causes of back pain after the procedure, especially when it is followed by neurological deficits which epidural hematoma is one of the culprits.^9^

The anatomy of the spinal and pelvic venous plexus is interrelated with a large anastomosis present in the venous trunk. The anterior and median posterior veins empty into the radicular veins, which subsequently drain into the vertebral plexus (both para‐ and intervertebral plexus). The final destinations are the azygous and pelvic veins.^10^ Our patient might have collaterals between the pelvic and spinal veins due to long‐term occlusion in the iliac veins. So, it is more likely that these vessels were injured during the procedure.

Both epidural and subdural spinal hematoma has been reported in the literature and it is challenging to differentiate between them based on MRI, therefore the surgeon should keep both in mind. In a case report by Qiyang Xu et al^11^, spontaneous spinal subdural hematoma happened after mechanical thrombectomy and catheter‐directed thrombolysis.

Spinal epidural hematoma was reported as an early complication of common iliac vein stenting by HS Kwak et al^12^ In their report, the patient presented the symptoms 4 h after the procedure. MRI showed SEH between T11 and L2, which led to surgical laminectomy. Unfortunately, the patient had partial hemiplegia even after 7 months. In our report, although after 3 months the function of the upper extremities was improved, the lower extremities still had a weakness.

The extent of the symptoms of SEH is related to the location and severity of the hematoma. In this case, acute severe back pain and progressive ascending paralysis were the main complaints of the patient. Late diagnosis and delayed surgery may cause devastating neurological sequelae in patients and the time between symptoms onset and surgical decompression is the main factor determining the outcome.^4^

## 4 CONCLUSION

It is crucial to check the wiring of the common iliac vein and the IVC in the lateral view to avoid spinal canal invasion. Inappropriate wiring of the para‐spinal space, the operator's unfamiliarity with the procedure complications, and delayed surgery due to late diagnosis can lead to this life‐threatening situation. Finally, although back pain is a frequent complaint after iliac vein stenting, procedure‐related complications should always be ruled out especially when there is radicular pain in the lower extremities accompanied by neurologic impairments.

## CONFLICT OF INTEREST

None declared.

## AUTHOR CONTRIBUTIONS

YJ: Angiography and venography, and scientific supervision. MEB: Scientific supervision. AA: Writing—original draft and writing—review and editing. ST: Search. KH: Writing—original draft and writing‐review and editing. HJ: Scientific supervision.

## ETHICAL APPROVAL

The protocol of this study is in line with the 2013 Helsinki declaration and was approved by the Ethics Committee of Tehran University of Medical Sciences. An informed consent was taken from the patient.

## CONSENT STATMENT

Published with written consent of the patient.

## Data Availability

The data that support the findings of this study are available from the corresponding author upon reasonable request.
